# Mechanosignaling via Integrins: Pivotal Players in Liver Fibrosis Progression and Therapy

**DOI:** 10.3390/cells14040266

**Published:** 2025-02-12

**Authors:** Aigul Sharip, Jeannette Kunz

**Affiliations:** 1 Department of Biomedical Sciences, Nazarbayev University School of Medicine, Astana 020000, Kazakhstan; aigul.sharip@nu.edu.kz; 2 Laboratory of Bioinformatics and Systems Biology, National Laboratory Astana, Astana 020000, Kazakhstan

**Keywords:** liver fibrosis, hepatic stellate cell, mechanotransduction, extracellular matrix, integrin, Hippo pathway, YAP, ROCK, MRTF, TGF-beta, therapeutic compounds

## Abstract

Liver fibrosis, a consequence of chronic liver injury, represents a major global health burden and is the leading cause of liver failure, morbidity, and mortality. The pathological hallmark of this condition is excessive extracellular matrix deposition, driven primarily by integrin-mediated mechanotransduction. Integrins, transmembrane heterodimeric proteins that serve as primary ECM receptors, orchestrate complex mechanosignaling networks that regulate the activation, differentiation, and proliferation of hepatic stellate cells and other ECM-secreting myofibroblasts. These mechanical signals create self-reinforcing feedback loops that perpetuate the fibrotic response. Recent advances have provided insight into the roles of specific integrin subtypes in liver fibrosis and revealed their regulation of key downstream effectors—including transforming growth factor beta, focal adhesion kinase, RhoA/Rho-associated, coiled-coil containing protein kinase, and the mechanosensitive Hippo pathway. Understanding these mechanotransduction networks has opened new therapeutic possibilities through pharmacological manipulation of integrin-dependent signaling.

## 1. Introduction

Fibrotic diseases are a leading cause of mortality in industrialized nations and often arise from chronic injuries, autoimmune reactions, toxin exposure, inflammation, or infection [1,2]. These conditions are characterized by excessive deposition of the extracellular matrix (ECM), which disrupts normal tissue architecture and function [3,4,5], potentially leading to organ failure and death [5,6]. Current therapeutic strategies predominantly focus on the underlying triggers rather than the fibrotic process itself [7,8,9,10,11]. While antiviral treatments can successfully manage virus-induced liver fibrosis, nonviral fibrosis remains difficult to treat, underscoring the urgency of discovering new therapeutic targets that disrupt the fundamental mechanisms of fibrotic disease [12,13].

At the cellular level, fibrosis progression is driven by mechanotransduction, the conversion of mechanical forces into biochemical signals, a process primarily mediated by integrins. During fibrosis, ECM accumulation stiffens tissues, which, in turn, enhances integrin signaling in a feedforward loop that further drives ECM production. These mechanical feedback loops operate through the RhoA/Rho-associated, coiled-coil containing protein kinase (ROCK) signaling axis, the Hippo pathway, and integrin-dependent activation of latent transforming growth factor beta (TGF-β), among others. Although many integrin subtypes contribute to fibrosis, αv and β1 integrins, in particular, exert key influences across multiple organs. However, their ubiquitous expression complicates direct therapeutic targeting. As a result, the targeting of downstream effectors of integrin-mediated mechanotransduction may offer more selective antifibrotic therapeutic strategies.

This review explores the primary mechanotransductive signaling networks that drive liver fibrosis, emphasizing how mechanical cues such as increased tissue stiffness activate hepatic stellate cells (HSCs), the principal ECM-producing cells in the liver. We highlight how HSCs sense and respond to mechanical forces through integrin-based adhesion complexes and cytoskeletal remodeling, which, in turn, promote the transdifferentiation of HSCs into myofibroblasts, leading to dysregulated collagen deposition and fibrotic scar formation. We further discuss the key integrin-dependent mechanosignaling pathways that drive the progression of liver fibrosis. Finally, we summarize current therapeutic strategies targeting these mechanotransduction pathways, focusing on emerging small-molecule inhibitors and biologics designed to disrupt integrin-dependent mechanosignaling. Some of these targeting strategies have already shown promise in preclinical and clinical studies for mitigating excessive collagen deposition and attenuating fibrosis progression, and they offer significant potential in preventing or reversing liver fibrosis.

## 2. Overview of Liver Fibrosis

### 2.1. Etiologies of Liver Injury and Disease Mechanisms

**Figure 1:**
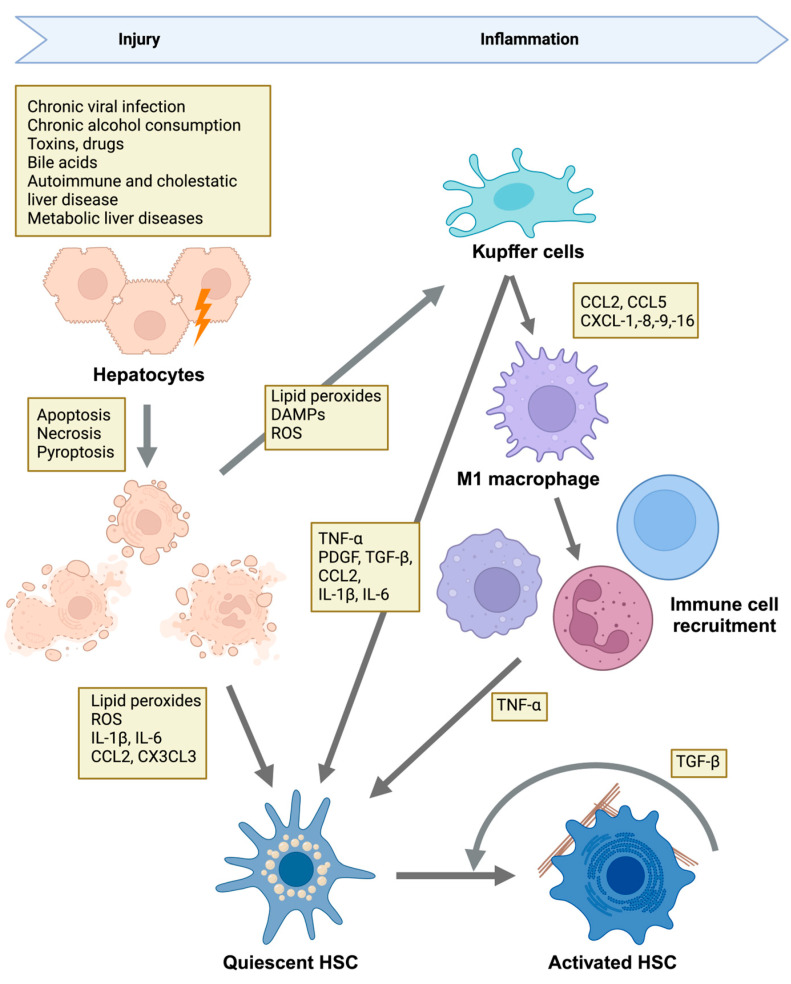
Overview of the cellular mechanism of HSC activation in the liver. Chronic injury to hepatocytes caused by infections, drugs, toxins, alcohol abuse, or metabolic liver disease leads to hepatocyte death and the release of DAMPs, apoptotic bodies, and ROS. These signals activate quiescent HSCs, stimulate resident Kupffer cells, and recruit additional immune cells, setting off complex, multidirectional interactions that promote the transdifferentiation of HSCs into proliferative, ECM-producing myofibroblasts. Kupffer cells and recruited macrophages respond to DAMPs by producing a wide array of proinflammatory cytokines and chemokines. This proinflammatory milieu helps recruit both innate and adaptive immune cells, amplifying profibrogenic pathways throughout the liver. In particular, Kupffer cells secrete IL-6 and TNFα, both of which are strongly proinflammatory. TNFα also induces hepatocyte apoptosis and neutrophil activation, which further supports HSC survival and proliferation. Certain chemokines, such as CCL2 and CCL5, play pivotal roles in immune cell recruitment. CCL2 activates Kupffer cells in an autocrine manner while also inducing proinflammatory gene expression in HSCs; both CCL2 and CCL5 help recruit additional immune cells. Moreover, CCL5 itself recruits and activates HSCs. Kupffer cells also increase the survival of activated HSCs by releasing PDGF, a primary mitogen for HSCs, and TGFβ. Once activated, HSCs secrete latent TGFβ, establishing an autocrine positive feedback loop that maintains their activated state. HSCs are recognized as the predominant source of myofibroblasts in liver fibrosis, producing up to 90% of the ECM in this context. The ongoing release of proinflammatory factors, growth factors, and cytokines combined with autocrine stimulation by TGFβ ultimately drives HSC activation, ECM deposition, and the progression of fibrosis. Abbreviations: CCL, C-C motif chemokine ligand; CX3CL3, C-X3-C motif chemokine ligand 1; DAMP, damage-associated molecular pattern; ECM, extracellular matrix; HSC, hepatic stellate cell; IL, interleukin; PDGF, platelet-derived growth factor; ROS, reactive oxygen species; TGF-β, transforming growth factor beta; TNF-α, tumor necrosis factor-alpha. The figure was generated using biorender.com, accessed on 14 January 2025.

Liver fibrosis is an aberrant wound-healing response characterized by the excessive deposition of ECM components, primarily collagen. This pathological ECM deposition results from chronic liver injury and the subsequent persistent activation of inflammatory and fibrogenic processes. Such injuries may result from viral hepatitis B and C (HBV, HCV) infection, metabolic disorders (e.g., nonalcoholic fatty liver disease [NAFLD], nonalcoholic steatohepatitis [NASH], metabolic-associated steatohepatitis [MASH]), alcoholic liver disease (ALD), and cholestatic conditions (e.g., primary sclerosing cholangitis [PSC], primary biliary cholangitis [PBC]) [14,15]. These chronic insults cause hepatocyte death through apoptotic, necrotic, or necroptotic pathways [16,17] and release reactive oxygen species (ROS), inflammatory cytokines, and damage-associated molecular patterns (DAMPs). DAMPs, in turn, activate Kupffer cells, resident liver macrophages, and other recruited inflammatory cells (Figure 1). These cells then secrete profibrotic factors such as TGF-β, platelet-derived growth factor (PDGF), chemokines, and interleukins (ILs) [17,18], which induce and amplify the activation of HSCs (Figure 1) [19,20,21,22].

While the triggers and inflammatory cues may vary on the basis of the underlying etiology, they promote connective tissue deposition in the hepatic parenchyma by converging on the activation and transdifferentiation of HSCs into myofibroblast-like cells, which produce excessive amounts of ECM proteins.

Thus, viral hepatitis (HBV, HCV) is characterized by chronic viral replication and an ongoing immune response that drives the secretion of cytokines (e.g., TNF-α, IL-1β) and chemokines, stimulating HSCs toward a profibrotic phenotype [15,23]. Repeated cycles of hepatocyte injury compound fibrogenic signals via DAMP release, reinforcing inflammation and HSC activation.

In metabolic disorders (MASH, NAFLD, and NASH), insulin resistance, hyperglycemia, and lipotoxic metabolites induce hepatocyte stress and activate Kupffer cells, promoting the secretion of profibrotic mediators [15,23]. These mediators sensitize HSCs to TGF-β and other cytokines, upregulate specific integrin subunits on HSCs and endothelial cells, and increase mechanical stress within the liver, perpetuating integrin-driven fibrogenic loops.

In ALD, excessive and prolonged alcohol intake results in the production of acetaldehyde and ROS, triggering hepatocyte damage, neutrophil infiltration, and macrophage activation [23]. This increases TGF-β release and HSC stimulation, leading to increased collagen deposition and impaired matrix degradation that culminates in cirrhosis.

Under cholestatic conditions (PBC/PSC), toxic bile acids injure cholangiocytes, creating a fibrotic niche around bile ducts. The stressed cholangiocytes secrete fibrogenic cytokines and growth factors, driving the activation of HSCs and immune cell recruitment [15,23]. Additionally, integrins such as αvβ6, which are highly expressed on cholangiocytes under cholestatic stress, promote local activation of latent TGF-β, amplifying SMAD-dependent fibrogenic pathways [15,23]. This interplay between cholangiocytes, HSCs, and ECM components underlies the distinct periductular fibrosis characteristic of cholestatic diseases.

Thus, regardless of etiology, the transformation of quiescent HSCs into activated myofibroblasts represents a critical event in liver fibrosis progression.

### 2.2. HSCs Are Responsible for Pathological ECM Deposition

HSCs are derived from various sources, including resident fibroblasts, epithelial cells undergoing epithelial–mesenchymal transition (EMT), and bone marrow-derived fibrocytes [19]. HSCs are perisinusoidal cells residing within the subendothelial space situated between hepatocytes and sinusoidal endothelial cells [19]. They constitute approximately 15% of the resident cells in the healthy human liver and predominantly account for collagen deposition (approximately 90%) in the fibrotic liver [23]. The cellular processes of HSCs extend throughout the space of Disse, positioning them to closely interact with hepatocytes, endothelial cells, and nerve endings [23,24]. Moreover, as described above, HSCs play an important role in initiating the immune response by engaging with Kupffer cells and immune cells. In addition, HSCs support angiogenesis and contribute to the regulation of oxidative stress [23,24].

**Figure 2:**
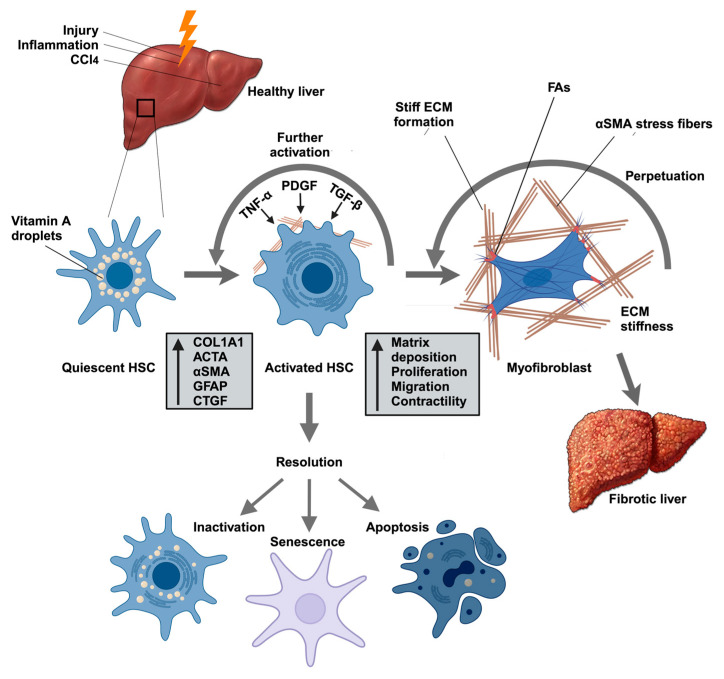
A schematic model of hepatic stellate cell (HSC) activation in liver fibrosis is illustrated. Hepatocellular injury or inflammation triggered by various insults activates quiescent, vitamin A-rich HSCs through signaling processes that involve cytokines such as TNF-α, TGF-β, and PDGF, among others. Upon activation, HSCs upregulate genes encoding pro-proliferative, cytoskeletal, and extracellular matrix components (e.g., *ACTA2*, encoding αSMA; *COL1A1*; and *FN1*, encoding fibronectin 1, *GFAP*, and *CTGF*), resulting in increased HSC proliferation and secretion of ECM. Liver fibrosis is often considered a reversible condition if the causative agents are eliminated and HSC activation is curtailed. Potential mechanisms for reversing HSC activation include returning to an inactivated state, entering senescence, or undergoing apoptosis as part of the resolution of the wound-healing response. However, chronic or repeated injury can drive sustained HSC activation, leading to excessive ECM deposition and hyperactive mechanosignaling. This creates a self-perpetuating, feedforward cycle in which stress fiber and focal adhesion formation heighten HSC contractility, fueling further activation and transdifferentiation into proliferative, fibrogenic myofibroblasts. These myofibroblasts ultimately drive fibrosis development and progression. Abbreviations: COL1A1, collagen type I alpha 1; CTGF, connective tissue growth factor; ECM, extracellular matrix; FN1, fibronectin 1; GFAP, glial fibrillary acidic protein; HSC, hepatic stellate cell; PDGF, platelet-derived growth factor; SMA, smooth muscle actin; TGF-β, transforming growth factor beta; TNF-α, tumor necrosis factor-alpha. The figure was generated using biorender.com, accessed on 8 January 2025.

Under normal conditions, HSCs maintain a quiescent state, functioning primarily as vitamin A storage cells (Figure 2) [24]. HSC activation occurs in distinct phases. During the initiation phase, HSCs lose their vitamin A storage capacity and become increasingly responsive to cytokines and growth factors, undergoing phenotypic changes that increase their contractility [24]. Activated HSCs also contribute to the recruitment of additional inflammatory cells to the wound area [24]. The subsequent perpetuation phase is characterized by increased proliferation and migration, enhanced ECM production (especially that of collagen), the acquisition of contractile properties, and the release of TGF-β and cytokines (Figure 2) [24,25,26]. Activated Kupffer cells, macrophages, and other inflammatory cells further release cytokines, such as TGF-β, PDGF, and tumor necrosis factor-alpha (TNF-α), that drive HSC activation, thus amplifying the fibrogenic response (Figure 1 and Figure 2) [23,24]. These cytokines stimulate the proliferation, migration, and differentiation of HSCs, which allows them to move to the site of injury and secrete ECM proteins, leading to wound repair.

Upon regeneration of healthy tissue, activated myofibroblasts are eliminated through apoptosis or senescence, or they revert to an inactive phenotype [27]. This results in the resolution of the fibrotic response [28,29]. Concurrently, ECM homeostasis is restored as matrix metalloproteinases (MMPs) break down ECM components.

If the injurious stimulus is eliminated (e.g., by antiviral therapy for HBV/HCV) or healthy tissue is restored, these activated myofibroblasts can undergo apoptosis, senescence, or reversion to a quiescent phenotype, promoting fibrosis resolution [28,29]. Because activated HSCs also exacerbate inflammation by releasing cytokines, chemokines, and growth factors, which then recruit and activate immune cells, such as T cells, B cells, and macrophages [30,31], the resolution of inflammation is, thus, equally essential for the completion of the repair process. As inflammation subsides, the secretion of proinflammatory cytokines decreases, which diminishes myofibroblast activity [29]. This decrease in activity leads to a reduction in the deposition of ECM proteins, aiding in the overall resolution of fibrosis. However, persistent injury drives self-reinforcing mechanotransductive feedback loops: as the ECM stiffens, it sustains integrin signaling in HSCs, accelerating excessive collagen deposition and enabling further tissue stiffening [20,32]. Disrupting these mechanical feedback mechanisms is now seen as a pivotal approach to treating liver fibrosis.

### 2.3. Targeting HSC Activation to Reversal of Liver Fibrosis

Current evidence suggests that early-stage fibrosis may be reversible, whereas advanced cirrhosis typically is not [29]. Resolution of fibrosis requires several key elements: removal of the underlying injury stimulus, HSC deactivation or elimination, enhanced ECM degradation, and resolution of inflammation. Conversely, factors driving progression include persistent injury or inflammation, sustained mechanical activation, impaired matrix degradation, and chronic HSC activation. Studies have shown that, if the root cause of fibrosis is addressed and the natural mechanisms for ECM degradation operate effectively, fibrotic tissue could be replaced with healthy tissue over time. For example, antiviral therapies for chronic hepatitis B and C infections not only halt disease progression but also promote the reversal of fibrosis or cirrhosis by reducing inflammation [7,8,9,10,11,33]. Therefore, therapeutic targeting of HSC activation may represent a promising antifibrotic strategy.

As described above, deactivation of HSCs can occur through apoptosis, senescence, or reversion to a quiescent phenotype. Accordingly, Troeger et al. reported that the induction of HSC apoptosis in mice led to decreased fibrosis and improved liver function [34]. Similarly, Kisseleva et al. reported that, during recovery from carbon tetrachloride (CCl_4_)- and alcohol-induced liver fibrosis, activated myofibroblasts either undergo apoptosis or revert to an inactivated state [28]. Additionally, targeting key signaling pathways involved in HSC activation has shown efficacy in reducing fibrosis. Inhibiting TGF-β signaling, one of the key fibrogenic pathways, can attenuate HSC activation and ECM production [29].

The complex interactions among cellular responses, mechanical forces, and matrix remodeling that occur in response to injury and during healing highlight additional potential therapeutic targets [27]. Given their central role in mechanosensing and signal integration, integrins present promising therapeutic targets. Strategies to modulate integrin activity or interfere with their nuclear signaling could reduce the mechanosensitive feedback driving fibrosis progression. For example, Henderson et al. demonstrated that targeting αv integrins reduced fibrosis in multiple organ models by interrupting the activation of TGF-β and downstream fibrogenic processes [35]. Furthermore, approaches that target the broader fibrogenic network within the liver, such as those aimed at reducing ECM stiffness, either through increased matrix degradation or inhibition of ECM synthesis, might help disrupt the integrin-mediated mechanical feedback loop. The following sections will examine specific molecular pathways, with a particular emphasis on integrin-mediated mechanotransduction, that may serve as therapeutic targets for liver fibrosis.

## 3. Integrin Signaling in Liver Fibrosis

Integrin-mediated interactions between cells and the ECM play a central role in fibrosis development, particularly in the transformation of quiescent fibroblasts into pathologically active myofibroblasts. While the initial triggers of this transformation remain incompletely understood, the crucial role of integrin signaling in this process is well established.

### 3.1. Overview of Integrin Signaling

**Figure 3:**
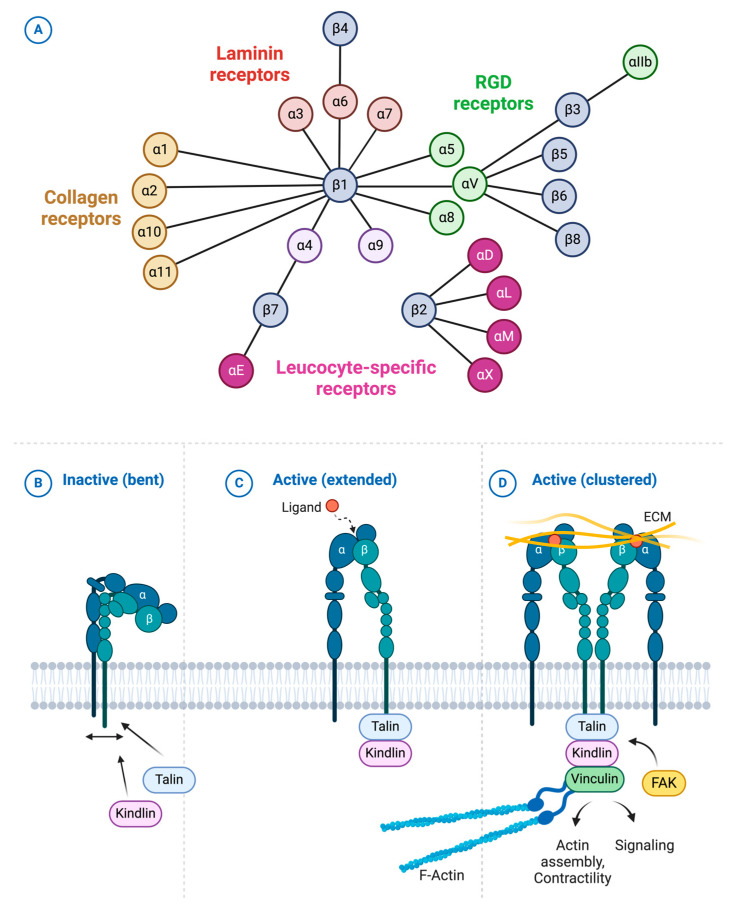
(**A**) Classification of integrins. Integrins are heterodimers composed of one α subunit and one β subunit. There are 24 different integrin heterodimers formed through combinations of 18 α and 8 β subunits. On the basis of the identity of their main ligands, integrins can be classified into four main subgroups. RGD-binding integrins (α3β1, αv β1, αv β3, αvβ5, αvβ6, αvβ8, and αIIβ3) interact with proteins containing the RGD peptide, such as fibronectin, vitronectin, fibrinogen, and thrombospondin-1. Collagen-binding integrins (α1β1, α2β1, α10β1, and α11β1) bind to both fibrillar and nonfibrillar collagens. Laminin-binding integrins (α3β1, α6β1, α7β1, and α6β4) interact with laminin-containing basement membranes. In addition, seven members of the integrin family, including β2 integrins, are expressed predominantly on the surface of leukocytes. (**B**–**D**) Key conformational states of integrins: (**B**) bent (inactive), (**C**) extended-open (active), and (**D**) extended-clustered. The structural components of integrins—the large extracellular domain, the transmembrane segment, and the short cytoplasmic tail—are crucial for their signaling processes. The extracellular domain interacts with ECM ligands, initiating outside-in signaling that propagates through the transmembrane segment to the cytoplasmic tail. This activation leads to the extended-open configuration of integrins and the subsequent recruitment of signaling and adaptor proteins to the cytoplasmic tails of integrin subunits. The assembly of signaling complexes at the cell membrane promotes integrin clustering and focal adhesion formation. Abbreviations: RGD, arginine–glycine–aspartic acid. FAK, focal adhesion kinase. The figure was generated via biorender.com, accessed on 13 January 2025.

Integrins constitute a diverse family of cell surface receptors that serve as primary mediators of cell–ECM adhesion and mechanotransduction. These receptors exist as heterodimers formed through noncovalent interactions between 18 α-subunits and 8 β-subunits, generating 24 distinct combinations (Figure 3A) [36].

This diversity enables precise control of cell–ECM interactions, with different integrin heterodimers showing specificity for distinct ECM components. On the basis of their ligand binding preferences and expression patterns, integrins can be categorized into four main families: Arg-Gly-Asp (RGD) receptors, collagen receptors, laminin receptors, and leukocyte-specific integrins (Figure 3A).

The functional versatility of integrins stems from their ability to undergo significant conformational changes in response to both extracellular and intracellular signals. Upon ligand binding, integrins shift from a low-affinity state to a high-affinity state through complex structural rearrangements (Figure 3B–D) [36]. This activation process not only enhances their binding to ECM components but also triggers the clustering of integrin molecules on the cell surface, increasing their signaling capacity [36]. The dual binding capability of integrins, which engage ECM components extracellularly while connecting to the cytoskeleton intracellularly, makes them uniquely suited to transmit mechanical signals across the plasma membrane.

This mechanical coupling enables bidirectional signal transmission. In outside-in signaling, ECM engagement triggers conformational changes that initiate the assembly of focal adhesion complexes, multiprotein assemblies that link the ECM to the cellular cytoskeleton [36,37]. This linkage facilitates the transduction of mechanical signals into the cell. Conversely, inside-out signaling allows intracellular cues to modulate the affinity and avidity of integrin extracellular domains for their ECM ligands, providing precise control over cell–ECM interactions. This process is mediated by intracellular signals that induce conformational changes in the cytoplasmic tail, which are transmitted through the transmembrane segment to the extracellular ligand-binding domain [36].

The engagement of specific ECM components, including laminin, fibronectin, collagen, and vitronectin, by their integrin receptors induces the recruitment of signaling and adaptor proteins to integrin tails, leading to the assembly of nascent focal adhesion contacts [38]. These cell‒matrix contacts trigger the polymerization of F-actin, which, in turn, promotes the stabilization and clustering of integrins on the cell surface, inducing the maturation of focal contacts to focal adhesions [39,40,41,42]. Focal adhesions link integrins to the actin cytoskeleton, generating contractile tension crucial for mechanotransduction. Through these adhesive structures, integrins sense changes in matrix composition and stiffness, triggering signaling cascades that collectively maintain tissue homeostasis [38,40,42,43,44].

Depending on the integrin involved, these include pathways regulated by focal adhesion kinase (FAK), integrin-linked kinase (ILK), the Rho/ROCK and Hippo pathways, as well as classical signal transduction pathways such as the mitogen-activated protein kinase (MAPK) and phosphoinositol 3-kinase (PI3K) pathways [36]. The complexity of integrin signaling is further enhanced by the cooperation of integrins with growth factor receptors and other cell surface molecules, enabling the integration of mechanical and biochemical signals into integrated cellular responses.

Through these signaling networks, integrins regulate a remarkably diverse array of cellular functions. They not only control basic cellular processes such as adhesion and migration but also influence cell survival, proliferation, polarity, and differentiation. In the context of tissue homeostasis, integrins play essential roles in maintaining structural integrity and orchestrating appropriate responses to injury by coordinating immune responses with tissue remodeling. The central role of integrins in these fundamental cellular processes explains why their dysregulation contributes to various pathological conditions, including fibrosis [38,45,46].

### 3.2. Roles of Integrins in HSC Activation

In liver fibrosis, integrins drive disease progression by directly activating survival and profibrotic signaling in HSCs and enabling the local activation of latent TGF-β within fibrotic niches. These processes reinforce a feedforward loop of stiff ECM, sustained integrin activation and clustering, and myosin-dependent cytoskeletal tension that further supports collagen deposition [47]. This mechanical feedback loop amplifies fibrogenic responses by triggering the activation, proliferation, and differentiation of HSCs.

HSCs express multiple integrins that bind to matrix proteins such as fibronectin (α5β1), vitronectin (αvβ3), and type I collagen (α1β1, α2β1). For example, α1β1- and α2β1-mediated interactions not only anchor HSCs within the space of Disse but also provide mechanical feedback that influences the activation state of HSCs by allowing the cells to respond to changes in matrix stiffness. The resulting fibrogenic responses are amplified, at least in part, through the action of αv integrins. These integrins, most prominently, play key roles in fibrogenesis by modulating the activation of latent TGF-β and downstream SMAD-dependent profibrotic signaling. Additionally, αvβ3 integrin-dependent survival signals via activation of the PI3K/Akt pathway contribute to the sustained presence of active, fibrogenic HSCs within fibrotic liver tissue, thereby promoting ongoing scar formation and tissue remodeling.

### 3.3. Other Disease-Specific Roles of Integrins in Liver Fibrosis

In addition to their roles in HSC activation, integrins also bridge inflammation and fibrosis. For example, integrins, especially those expressed in immune cells, endothelial cells, cholangiocytes, and HSCs, play central roles in modulating fibrotic progression by controlling immune cell infiltration and cytokine activation [17,23]. Notably, different etiologies, such as biliary disorders, chronic viral hepatitis, and metabolic-associated steatohepatitis, employ both unique and shared integrin–ECM interactions for the recruitment and retention of inflammatory cells [17,23]. Understanding these disease-specific inflammatory patterns may aid in the development of more specific and effective therapeutic strategies to block fibrosis progression.

In cholestatic liver diseases, cholangiocyte injury leads to the recruitment of inflammatory cells, particularly T cells and monocytes, around the portal tracts [23]. Injured, reactive, cholangiocytes, which play pivotal roles in driving fibrogenesis in biliary liver disorders and in advanced stages of liver fibrosis of various etiologies [17], upregulate adhesion molecules, including integrins, that facilitate the adhesion and transmigration of immune cells into portal areas [48]. For example, integrin α4β1-mediated recruitment of mononuclear cells from periportal sinusoids is heightened under cholestatic conditions. Additionally, integrin-mediated activation of latent TGF-β1 via αvβ6 and αvβ8 expressed on reactive cholangiocytes can further amplify fibrogenic signaling toward periportal fibroblasts [48]. Thus, in biliary disease, portal inflammation characterized by integrin-dependent trafficking of immune cells and integrin-mediated TGF-β activation sets the stage for biliary-type fibrosis.

While integrin expression patterns may be similar among different etiologies, the nature of the immune infiltrate in viral hepatitis is dominated by antiviral cytotoxic T cells and supported by Kupffer cell activation [17,23]. Virus-infected hepatocytes serve as initiators for the predominantly T-cell–driven inflammatory response. Integrin-expressing T lymphocytes (e.g., LFA-1/αLβ2 and VLA-4/α4β1) and NK cells accumulate in the liver parenchyma to clear infected cells. This cytotoxic and proinflammatory environment, in turn, activates Kupffer cells and HSCs. Integrins such as αV integrins expressed on Kupffer cells and sinusoidal endothelial cells can also modulate the stiffness of the ECM and the activation of latent TGF-β, driving HSC activation. Over time, these sustained antiviral responses and persistent integrin-dependent cell‒cell and cell‒ECM interactions promote perisinusoidal fibrosis, ultimately leading to cirrhosis.

MASH-related fibrosis typically arises from a metabolic and lipotoxic milieu rather than an external pathogen. The release of free fatty acids, oxidative stress, and DAMPs from steatotic hepatocytes initiates a mixed inflammatory response characterized by infiltration of macrophages (including recruited monocyte-derived macrophages) and neutrophils [17,23]. Macrophages express integrins such as αMβ2 and αv-containing integrins that interact with ECM components, guiding their localization and retention in the injured liver. This macrophage infiltration is often accompanied by integrin-mediated activation of latent TGF-β and the subsequent myofibroblastic transformation of HSCs. The metabolic nature of MASH-related injury also fosters an environment where integrin-mediated interactions not only perpetuate inflammation but also shape the ECM composition, leading to elevated fibrillar collagen and altered laminin networks. Thus, integrins in MASH connect metabolic injury to progressive fibrosis by directing macrophage behavior, TGF-β activation, and ECM remodeling.

In ALD, the initial injury to hepatocytes from ethanol metabolism and its byproducts, such as acetaldehyde, triggers robust neutrophil and macrophage infiltration [17,23]. Integrins are key in mediating the adhesion and transmigration of these leukocytes across sinusoidal endothelial cells. Integrin αLβ2 (LFA-1) and αMβ2 (Mac-1) interact with their ligands (ICAM-1 on endothelial cells) and are crucial for the formation of the inflammatory infiltrate typical of alcoholic hepatitis. Like other etiologies, activated macrophages and HSCs utilize integrins (e.g., αv integrins) to control TGF-β activation and matrix deposition.

Thus, by targeting specific integrins, particularly αv integrins, and/or the integrin-dependent activation of latent TGF-β (for example, by blocking αvβ6 and αvβ8), it may be possible to develop more effective targeted therapeutic strategies that attenuate both HSC activation and inflammation.

## 4. Integrin Regulation of Signaling Cascades in Liver Fibrosis

Integrin-mediated mechanotransduction in HSCs during fibrogenesis operates through several pathways that form an interconnected mechanosensitive network that amplifies and sustains fibrogenic responses. Among these pathways, the Hippo pathway, the Rho/ROCK signaling axis, and the TGF-β receptor pathway play key roles in mechanotransduction. These signaling cascades ultimately converge on several key transcriptional regulators, including myocardin-related transcription factor (MRTF), yes-associated protein (YAP), its paralog transcriptional *coactivator* with PDZ-binding motif (TAZ), and the suppressor of mothers against decapentaplegic (SMAD) proteins [49,50]. The activation of these factors enhances the expression of genes encoding ECM components, particularly collagens and alpha-smooth muscle actin (α-SMA), as well as various profibrotic mediators [3,4,49,50,51,52,53,54,55]. Below, we summarize the key components of these pathways and their effects on HSC activation and liver fibrosis.

### 4.1. Integrin-Mediated Mechanotransduction Through Rho/ROCK and MRTF

#### 4.1.1. The Core Rho/ROCK/MRTF Pathway

A critical downstream effect of integrin engagement in HSCs is the reorganization of the actin cytoskeleton. When integrins bind extracellular matrix ligands such as collagen or fibronectin, they activate signaling cascades that ultimately lead to RhoA GTPase activation. In turn, RhoA drives focal adhesion and stress fiber formation by promoting integrin clustering and actin polymerization, increasing cell contractility. The Rho/ROCK/MRTF signaling axis is a key pathway that relays these mechanical signals to the nucleus, thereby linking ECM stiffness to changes in gene expression.

**Figure 4:**
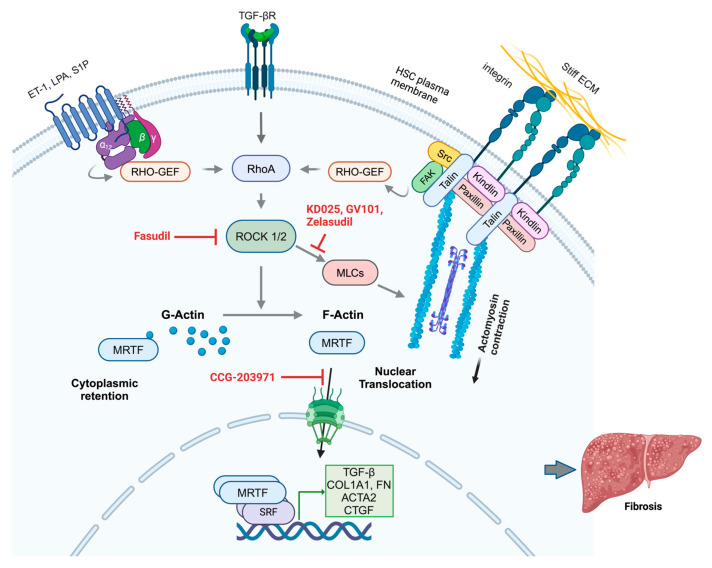
The Rho/ROCK/MRTF/SRF transcription pathway and its upstream regulation by the actin cytoskeleton in liver fibrosis. MRTFs (MRTF-A/MRTF-B) are transcriptional coactivators regulated by the ratio of F-actin to G-actin. Cytosolic G-actin binds MRTF masking a nuclear localization sequence. The activation of small RHAs by specific GEFs promotes F-actin polymerization via the RhoA effectors ROCK 1 and ROCK2, thereby reducing the cytosolic G-actin pool. This causes G-actin to dissociate from MRTF, unmasking the nuclear localization sequence, thereby allowing MRTF to translocate into the nucleus. MRTF binds the transcription factor SRF, forming the MRTF/SRF complex, which induces gene expression by binding SRE response elements. This, in turn, activates the transcription of genes encoding a subset of contractile proteins and fibrosis-associated proteins. Upstream activators of the pathway are biomechanical forces transduced by integrins, certain GPCRs, and TGF-β. Abbreviations: AngII, angiotensin II; COL1A1, collagen type I alpha 1; CTGF, connective tissue growth factor; ECM, extracellular matrix; ET-1, endothelin-1; FAK, focal adhesion kinase; FN1, fibronectin 1; HSC, hepatic stellate cell; LPA, lysophosphatidic acid; MLC, myosin light chain; RHO-GEF, Rho guanine nucleotide exchange factor; S1P, sphingosine-1-phosphate; ACTA2, smooth muscle actin (SMA); SRF, serum response factor; TGF-β, transforming growth transforming growth factor beta. The compounds that can influence some stages of the Hippo/YAP/TAZ pathway are highlighted in red. (Created with BioRender.com, accessed on 5 February 2025).

Recent evidence points to specific integrin heterodimers as major triggers of the RhoA/ROCK pathway. For example, upon binding fibronectin, α5β1 integrin recruits specialized guanine nucleotide exchange factors (GEFs) (e.g., GEF-H1/ARHGEF2, LARG/ARHGEF12) that stimulate RhoA activation, thereby promoting focal adhesion assembly under mechanical stress (Figure 4) [47,56,57,58]. Similarly, αvβ3 integrin forms mechanosensitive complexes with syndecans, recruiting adaptor proteins, such as syntenin and α-actinin, which facilitate force transmission and downstream signaling [47,59]. In addition to these ECM-based mechanical cues, chemical signals, such as ET-1, Ang II, sphingosine-1-phosphate (S1P), and lysophosphatidic acid (LPA), activate G protein-coupled receptors (GPCRs) to induce RhoA activation (Figure 4) [60,61,62,63,64,65,66,67,68]. Moreover, via activation of RhoA signaling, TGF-β can promote focal adhesion kinase (FAK) and the autophosphorylation of FAK at Tyr397, which plays a critical role in mechanotransduction and promotes focal adhesion formation, αSMA synthesis, and ECM production (Figure 4) [69].

The RhoA signaling pathway regulates several downstream effectors, most notably Rho-associated coiled-coil-containing protein kinase (ROCK), which consists of two isoforms, ROCK1 (also known as p160ROCK and ROKbeta) and ROCK2 (also known as Rho-kinase and ROKalpha), that regulate cytoskeletal dynamics and cellular contractility in a partially redundant manner. Activated RhoA enhances ROCK activity, leading to the phosphorylation of myosin light chains (MLCs), which are crucial for HSC contraction and migration (Figure 4) [62,66,70,71,72,73]. This pathway is also intricately linked to integrin signaling, which further activates RhoA in response to enhanced ECM stiffness, creating a positive feedback loop that perpetuates HSC activation and contributes to the progression of fibrosis.

The ultimate effectors in this signaling network are members of the MRTF family (MRTF-A/MKL1/MAL and MRTF-B/MKL2), which directly interact with the actin cytoskeleton. In their inactive state, MRTFs are sequestered in the cytoplasm (Figure 4) [74]. A critical step in MRTF activation and induction of the MRTF-dependent gene expression program is the shift in the intracellular ratio of globular actin (G-actin) to filamentous actin (F-actin). Under high-F-actin conditions, MRTF is released from G-actin–mediated sequestration in the cytoplasm and is free to enter the nucleus (Figure 4) [74].

Once in the nucleus, MRTF serves as a coactivator of serum response factor (SRF), a transcription factor that regulates genes involved in cytoskeletal dynamics and ECM production [74,75]. While initially characterized in smooth muscle cells, MRTF generally regulates fibroblast activation, including the activation of HSCs. MRTF, in complex with SRF, drives the expression of serum response element (SRE)-containing genes, including *ACTA2* (encoding αSMA), CTGF, integrins α2 and α11 (collagen binding), integrin αv (fibronectin and latent TGFβ1 binding), and the corresponding β-integrins β1, β3, and β5, as well as collagens, matrix metalloproteinases (MMPs), ILK, and TAZ [76,77]. In addition, MRTF can facilitate the transcription of genes by binding to other transcription factors, such as SMAD3 and TAZ, which induce transcription via alternative cis-elements. Therefore, MRTF signaling can amplify TGF-β-dependent or TAZ-dependent gene expression [78,79]. Thus, once activated, the MRTF/SRF pathway orchestrates a profibrotic transcriptional program that sustains ECM remodeling and HSC activation. Similarly, integrin-dependent MRTF activation increases cellular contractility [80], increasing mechanical tension within the fibrotic liver. This heightened tissue stiffness feeds back to intensify integrin signaling, closing the vicious cycle of progressive fibrosis.

#### 4.1.2. Experimental Evidence Linking the Rho/ROCK/MRTF/SRF Axis to Fibrosis

While the role of the Rho/ROCK/MRTF/SRF signaling cascade has been more extensively characterized in the context of kidney, cardiac, and lung fibrosis [81,82,83], the inhibition or genetic deletion of specific components within the signaling cascade has underscored the central role of this pathway in liver fibrosis. For example, pharmacological inhibition of ROCK1 with Y-27632 or ROCK2 with fasudil in hepatic stellate cells inhibited their activation [61,63,71,84,85,86], whereas ROCK inhibition in rodent models prevented liver fibrosis progression [66,84,85,87]. Furthermore, MRTF-A activity with the novel small molecule CCG-203971 resulted in dose-dependent suppression of HSC activation and liver fibrosis in vivo [50]. In line with these findings, MRTF-A–deficient knockout mice were resistant to CCl_4_-induced fibrosis [88,89]. Finally, conditional SRF deletion in HSCs attenuated both bile duct ligation (BDL)- and CCl_4_-induced liver fibrosis in mice [90].

These findings indicate that the Rho/ROCK/MRTF/SRF pathway is central to the transition of fibroblasts and HSCs to myofibroblasts, bridging environmental cues and transcriptional programs that drive fibrogenesis. By translating mechanical and biochemical signals into gene programs that drive myofibroblast activation, components of this pathway represent attractive therapeutic targets. A deeper understanding of integrin–MRTF/SRF signaling in liver fibrosis will aid in the development of more effective targeted therapies aimed at halting fibrotic progression.

### 4.2. The Hippo/YAP/TAZ Pathway

#### 4.2.1. The Hippo Core Signaling Cascade

**Figure 5:**
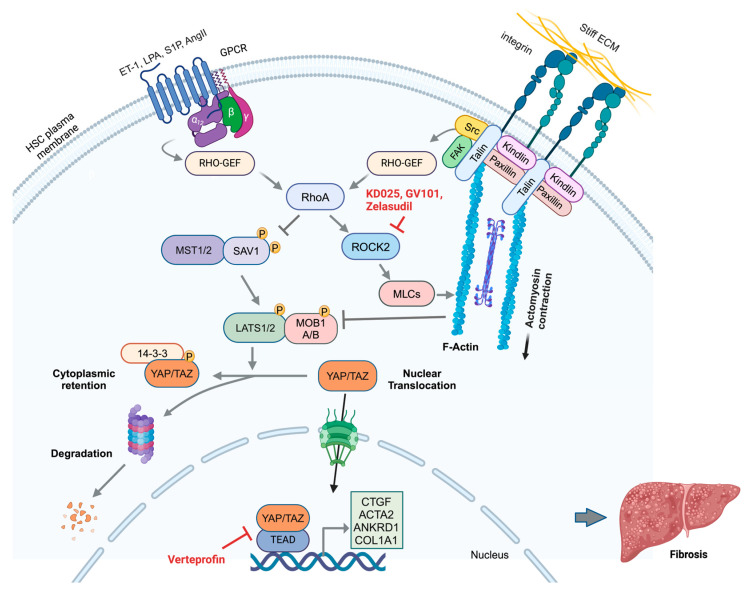
YAP-mediated gene expression in liver fibrosis. Simplified scheme showing the regulation of profibrotic gene expression by YAP during liver fibrosis and the attenuation of gene expression by the inhibitor verteprofin. When the Hippo pathway is activated, the active Hippo core kinase complex phosphorylates YAP at five serine residues, leading to either the cytoplasmic retention of YAP through interaction with 14-3-3 proteins or the degradation of YAP by the proteasome. Inactivation of the Hippo core kinase complex in response to initial tissue injury or mechanotransduction prevents YAP phosphorylation, allowing its nuclear translocation. Once in the nucleus, YAP forms a complex with TEAD transcription factors, leading to the upregulation of profibrotic genes, such as *CTGF*, *COL1A1*, *ACTA2*, and *ANKRD1*, among others. Treatment of HSCs or animal models with the FDA-approved drug verteprofin, which blocks the interaction between YAP and TEADs, inhibits YAP/TEAD-mediated profibrotic gene expression, HSC activation, and the progression of fibrosis. The precise mechanism leading to the activation of Hippo pathway activity in response to upstream signals during liver fibrosis remains to be fully determined. However, when cells encounter a rigid ECM, they respond by adjusting their cytoskeletal tension through integrin-mediated focal adhesions, which link the ECM to the intracellular F-actin cytoskeleton, involving kinases, such as Src and FAK, and adhesion proteins, such as vinculin, talin, and paxillin. Integrin-mediated mechanosignaling induces RhoA activation via RHO-GEFs, which triggers the reorganization of the cytoskeleton. RhoA, via the ROCK2-dependent regulation of myosin II motors, also promotes an increase in actomyosin tension. The increase in cell contractility, among other actin-dependent mechanisms, modulates YAP/TAZ nuclear translocation by inhibiting Hippo pathway signaling. YAP/TAZ then interact with TEAD transcription factors to promote target gene expression. Multiple other upstream inputs, such as GPCR signaling induced by ligands such as ET-1, Ang II, S1P, and LPA, can inhibit Hippo signaling via RhoA. Abbreviations: ACTA2, smooth muscle actin (SMA); AngII, angiotensin II; ANKRD1, ankyrin repeat domain 1; COL1A1, collagen type I alpha 1; CTGF, connective tissue growth factor; ECM, extracellular matrix; ET-1, endothelin-1; FN1, fibronectin 1; FAK, focal adhesion kinase; HSC, hepatic stellate cell; LPA, lysophosphatidic acid; MLC, myosin light chain; RHO-GEF, Rho guanine nucleotide exchange factor; S1P, sphingosine-1-phosphate; SRF, serum response factor; TGF-β, transforming growth transforming growth factor beta. The compounds that can influence some stages of the Hippo/YAP/TAZ pathway are highlighted in red. (Created with BioRender.com, accessed on 2 February 2025).

Originally characterized in *Drosophila melanogaster*, the Hippo pathway modulates organ size and regeneration, cancer, and fibrotic responses by regulating the phosphorylation status of Yes-associated protein (YAP) and its paralog TAZ [91,92]. The core Hippo pathway involves a kinase cascade in which macrophage stimulating 1/2 (MST1/2) and neurofibromatosis 2 (NF2), the mammalian homologs of Hippo and Salvador, activate the large tumor suppressor kinase 1/2 (LATS1/2)-MOB kinase activator 1A/B (MOB1A/B) complex (Figure 5). This complex phosphorylates YAP/TAZ, resulting in their sequestration in the cytoplasm by 14-3-3 proteins and/or subsequent degradation via the ubiquitin‒proteasome system (Figure 5). As a result, active Hippo pathway signaling prevents the nuclear translocation of YAP and TAZ and thereby inhibits their ability to induce gene expression, which promotes cell proliferation and survival (Figure 5).

When Hippo signaling is inactive, unphosphorylated YAP/TAZ translocates to the nucleus and binds to one of four closely related TEA domain (TEAD) transcription factors. YAP/TAZ–TEAD complexes associate with gene enhancers, recruit chromatin remodeling factors (e.g., BRD4), and modulate RNA polymerase II to regulate the transcription of profibrotic and proproliferative genes (Figure 5) [93,94,95].

#### 4.2.2. Mechanical Regulation of the Hippo Pathway

The Hippo pathway acts as a sensor of tissue and cellular integrity. Its activity is influenced primarily by changes in mechanical properties and overall cell shape. Consequently, various upstream components regulate Hippo signaling: integrin complexes, adherens junctions, and tight junctions serve as mechanical hubs that affect cell adhesion, morphology, and polarity [93,96,97] (Figure 5). The stiffness of the ECM and mechanotransduction via integrins are major upstream regulators of YAP/TAZ. The engagement of ECM ligands on stiff matrices induces integrin-dependent focal adhesion formation, RhoA activation, and actin stress fiber development [98,99] (Figure 5). Mechanical tension transmitted by actin suppresses Hippo kinases, permitting unphosphorylated YAP/TAZ to translocate into the nucleus and drive the expression of profibrotic genes, such as *COL1A1*, *ACTA2*, *ANKRD1* (ankyrin repeat domain 1), and *CTGF*, as well as genes that promote cell proliferation and survival (Figure 5) [93,96,97].

Several integrins and downstream signaling pathways have been linked to YAP/TAZ activation [40,99,100,101]. Constitutively active β1 integrin, for example, enhances YAP nuclear localization and transcription of profibrotic genes [55]. Furthermore, αv integrin influences YAP under mechanical loading [102] and during tumor invasion [103], whereas the targeted deletion of αv results in an attenuated response to mechanical loading [102]. Additionally, cell adhesion to fibronectin enhances YAP nuclear localization through the FAK-Src-PI3K pathway in a LATS1/2-dependent manner [104]. In the liver, Kitsugi et al. linked RGD integrins to HSC activation [105]. Furthermore, Martin et al. (2016) demonstrated that β1 integrin, through its association with the α11 subunit, activates HSC transdifferentiation into myofibroblasts by regulating the actomyosin cytoskeleton [106], affecting the ability of the cell to respond to biomechanical changes such as matrix stiffness. Conversely, the loss of β1 integrin led to reduced stress fiber formation and increased cytoplasmic sequestration of phosphorylated YAP [106]. This, in turn, abrogated the expression of profibrogenic YAP target genes, including *COL1A1*, *CTGF*, *ITGA11*, and *ITGB1*, and diminished HSC activation [106]. Collectively, these findings support a role for YAP in integrin-based signaling that perpetuates the fibroproliferative activation of myofibroblasts.

Therefore, by bridging mechanical forces and transcriptional control, integrin–YAP/TAZ crosstalk is integral to the self-perpetuating loop of tissue stiffening and HSC activation that characterizes advanced fibrosis. Consequently, therapies targeting integrin signaling or YAP/TAZ activity may disrupt these feedforward circuits and halt fibrosis progression [52].

#### 4.2.3. YAP/TAZ Activation Promotes HSC Activation and Fibrogenesis

In the context of the liver, the Hippo pathway has been implicated in controlling organ size and liver regeneration after injury, as well as in pathological conditions, including liver fibrosis and cancer [49,96,97,107].

In the healthy liver, Hippo pathway activity serves to restrain HSC activation and prevent excessive fibrogenesis. However, in response to acute tissue damage, the Hippo pathway is transiently inhibited, leading to YAP/TAZ nuclear translocation and activation of a gene expression program that promotes HSC activation, transdifferentiation, and regenerative ECM synthesis. When liver injury becomes chronic, hyperactivation of YAP/TAZ/TEAD-dependent transcription contributes to the fibrotic process by promoting excess ECM deposition, leading to scar formation and tissue stiffening. Thus, conditions that induce inflammation and fibrosis, such as NAFLD, can lead to chronically altered Hippo signaling, resulting in increased YAP expression and persistent hyperactivation of YAP-dependent gene expression.

In chronic liver injury models, YAP accumulates in the nucleus of HSCs after CCl_4_-induced damage or the induction of unilateral ureteral obstruction (UUO), triggering HSC activation and transdifferentiation into myofibroblasts [3,4,53]. In vitro studies further demonstrated that YAP accumulates in the nuclei of fibroblasts and HSCs in response to rigid matrices through focal adhesion-mediated intracellular force generation, promoting the expression of genes that support myofibroblast differentiation [52]. Conversely, growth on soft matrices or the detachment of cells from the ECM leads to the nuclear export of YAP/TAZ, preventing the activation and transdifferentiation of fibroblasts and HSCs [52].

YAP hyperactivation supports the excessive deposition of ECM, which, in turn, enhances integrin signaling and stress fiber polymerization, amplifying mechanotransductive signaling. Ultimately, this establishes a feed-forward loop in which YAP-induced matrix stiffening further enhances YAP activation, leading to ECM deposition, scar formation, and tissue stiffening, which are characteristic of fibrotic diseases [108].

Consistent with this notion, injecting human fibroblasts that overexpress constitutively active forms of YAP or TAZ into immunocompromised mice led to extracellular matrix accumulation and the development of fibrosis [53]. Conversely, the suppression of YAP expression in HSCs or the inhibition of YAP with verteporfin reduced the transcription of YAP target genes and blocked or reversed HSC activation [53,109,110]. Verteporfin treatment also attenuated CCl_4_- and BDL-induced liver fibrosis in mice [106], supporting findings by Mannaerts et al. and Bai et al., respectively [53,111]. Similar antifibrotic effects of verteporfin have been observed in other organ systems, such as UUO-induced renal fibrosis [54]. Notably, the mechanotransductive effects of YAP are not limited to fibrotic conditions: YAP is also required for cancer-associated fibroblasts to promote matrix stiffening, a process that is actomyosin- and Src kinase-dependent [108].

### 4.3. Integrin αv-Mediated Latent TGF-β Activation

#### 4.3.1. Molecular Mechanisms of Latent TGF-β Activation

TGF-β signaling is widely regarded as one of the key fibrogenic pathways driving HSC activation and ECM production. In a healthy liver, quiescent HSCs produce only a small amount of TGF-β, but its expression increases rapidly following liver injury [112]. Once HSCs are activated, they secrete TGF-β, establishing a positive feedback loop that further reinforces HSC activation [5,113]. Other cellular sources of TGF-β in the liver include liver sinusoidal endothelial cells, macrophages, and hepatocytes.

TGF-β is secreted as an inactive, latent complex (LLC) comprising latency-associated peptide (LAP) and latent TGF-β-binding protein (LTBP) [114,115,116]. The latent TGF-β stored in the ECM is activated and released through the action of integrins present on HSCs or cholangiocytes. During both acute and chronic liver injury, the LLC becomes anchored within the ECM through interactions between LTBP and ECM proteins (e.g., fibrillin or fibronectin). By tethering latent TGF-β to structural components of the ECM, the extracellular space effectively becomes a reservoir. This ensures that TGF-β can be rapidly mobilized and activated at specific sites where it is needed without being diffusely activated everywhere [117].

**Figure 6:**
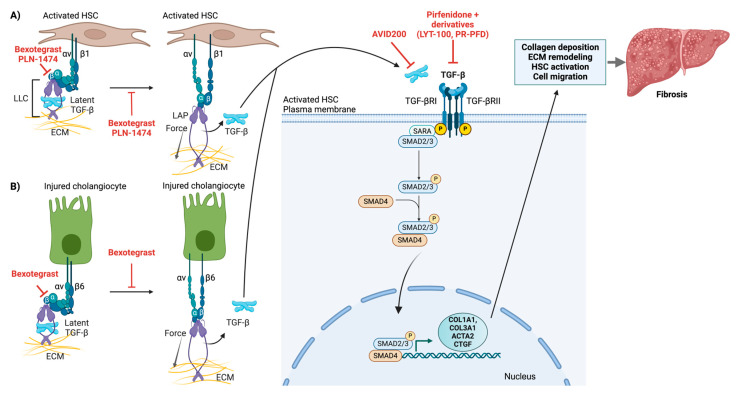
A model depicting systemic inhibition of canonical TGF-β signaling in the fibrotic liver by pirfenidone, pirfenidone derivatives, and the TGF-β trap AVID200, as well as the localized inhibition of latent TGF-β activation in the fibrotic niche by Bexotegrast (**A**,**B**), and PLN-1474 (**A**). Systemic and localized inhibition of canonical TGF-β signaling both attenuate the synthesis of profibrotic genes, such as *COL1A1*, *COL3A1*, and *ACTA2.* This affects HSC activation and transdifferentiation, as well as ECM remodeling, tissue stiffness, and invasive properties of myofibroblasts. Bexotegrast acts as a dual αVβ6 (present on activated HSCs) and αVβ1 (present on injured cholangiocytes) integrin inhibitor that attenuates integrin-dependent activation of latent TGF-β. PLN-1474 is a specific αVβ1 integrin inhibitor that also prevents latent TGF-β activation. Pirfenidone globally blocks the synthesis and activation of TGF-β. Only the canonical TGF-β signaling pathway is depicted. Abbreviations: ACTA2, smooth muscle actin (SMA); COL1A1, collagen type I alpha 1; COL3A1, collagen type III alpha 1; CTGF, connective tissue growth factor; ECM, extracellular matrix; HSC, hepatic stellate cell; LAP, Latency-Associated Peptide; LLC, large latent complex; SARA, anchor for receptor activation; SMAD, mothers against decapentaplegic homolog; TGF-β, transforming growth factor beta; TGF-βRI: transforming growth factor beta receptor type I; TGF-βRII: transforming growth factor beta receptor type II. The inhibitory compounds that can influence some stages of TGF-β activation and signaling are highlighted in red. (Created with BioRender.com, accessed on 5 February 2025).

There are two primary mechanisms for the activation of latent TGFβ in the fibrotic niche in the liver: proteolytic activation and integrin-mediated force-dependent activation [116,118]. In the protease-dependent mechanism, integrin binding facilitates TGF-β activation through the recruitment of specific metalloproteases, particularly MMP-14 and ADAMTS1, facilitating the activation of TGF-β through proteolytic cleavage of LAP [35,118]. This process often occurs in specialized membrane microdomains enriched in tetraspanins, which help organize the activation complexes. Force-dependent latent TGFβ activation involves multiple αv integrins (αvβ3, αvβ5, αvβ6, and αvβ8) transmitting cellular traction forces through the mechanical linkage between ECM-bound LTBP and the actomyosin cytoskeleton (Figure 6) [119,120,121]. This induces conformational changes in LAP, leading to the release of active TGF-β without the need for proteolytic cleavage.

These two mechanisms are not mutually exclusive and often work in concert. Mechanical force application can increase protease accessibility to LAP cleavage sites, whereas mechanical stress influences the spatial organization of activation complexes on the cell surface. The relative importance of each mechanism may vary depending on the cell type, matrix composition, tissue mechanical properties, and stage of disease progression. However, integrin-mediated activation of latent TGF-β is the predominant activation mechanism in the liver.

Recent structural studies have revealed that αv-containing integrins recognize specific conformational elements within the RGD motif of LAP, with αvβ6 and αvβ8 showing the highest binding affinity [122,123]. The efficiency of this force-dependent activation is influenced by extracellular matrix stiffness, which affects focal adhesion formation and force transmission [121,124]. The RhoA/ROCK signaling pathway regulates actomyosin contractility, thereby influencing TGF-β activation through this mechanism.

#### 4.3.2. Canonical TGF-β Signaling Pathway

TGF-β drives fibrosis progression from the initial stages of liver injury to the development of fibrosis and, ultimately, cirrhosis [113,125]. Among the five TGF-β isoforms identified in mammals, three (TGF-β1, TGF-β2, and TGF-β3) are expressed in humans [126]. In addition, other members of the TGF-β superfamily (such as activins and bone morphogenetic proteins) can activate this pathway. Pathway signaling is initiated by the binding of TGF-β to type II receptors (TGF-βRII). This interaction induces the formation of a heterotetrameric receptor complex by recruiting type I receptors (TGF-βRIs), which are also serine/threonine kinases. The type II receptor then phosphorylates and activates the type I receptor [127]. Once activated, TGF-βRI phosphorylates receptor-regulated SMAD2 and SMAD3 at specific serine residues in their C-terminal regions, a step crucial for their activation [127]. The phosphorylated SMAD2 and SMAD3 subsequently form a trimeric complex with SMAD4, which then translocates into the nucleus to function [127].

Once in the nucleus, the SMAD complex functions as a transcription factor by binding SMAD-specific DNA sequences (known as SMAD-binding elements) in the regulatory regions of target genes. To increase or repress transcription, the SMAD complex can collaborate with additional factors (such as coactivators, corepressors, and chromatin remodelers) that alter chromatin structure and recruit the transcriptional machinery.

In the context of fibrosis, the nuclear SMAD complex upregulates profibrogenic genes, such as those encoding collagen, fibronectin, and other extracellular matrix components [127]. It can also induce the expression of genes such as ACTA2 (α-SMA), which promotes the transition of fibroblasts into myofibroblasts. Through these actions, the TGF-β pathway, when deregulated, can drive the production of excessive ECM proteins, contributing to tissue fibrosis.

Pathway activity is regulated by inhibitory SMADs (such as SMAD6 and SMAD7), which antagonize SMAD2/3 activation and contribute to a negative feedback loop that maintains cellular homeostasis [127].

In addition to the canonical SMAD-dependent pathway, TGF-β can also activate noncanonical signaling via molecules such as Rho GTPases (e.g., RhoA) and MAPK cascades (including ERK and p38 MAPK). These alternative pathways mediate cellular responses, such as actin reorganization and contractility, independently of SMAD activation [127]. Furthermore, crosstalk with other signaling pathways (e.g., MAPK) can fine-tune both canonical and noncanonical TGF-β signaling [127].

#### 4.3.3. Evidence for Integrin-Mediated TGF-β Activation

The importance of integrin-mediated TGF-β activation in liver fibrosis has been demonstrated through various genetic approaches. Cell type-specific deletion studies have provided particularly compelling evidence. The conditional deletion of αv integrin in HSCs significantly inhibited liver fibrosis [35]. Global and conditional αv knockout led to reduced TGF-β activation, decreased fibrotic marker expression, and altered inflammatory responses.

Similarly, the deletion of αvβ1 integrin from myofibroblasts substantially suppressed fibrosis across multiple organs, including the liver, lung, and kidney [128,129,130,131].

Studies focusing on specific integrin subunits have further highlighted their roles in TGF-β activation. In activated HSCs, multiple αv integrins, including αvβ1, αvβ3, αvβ5, and αvβ8, are upregulated [131,132]. Studies have shown that the expression of these integrins increases during HSC activation and fibrosis progression [131,132]. Moreover, αvβ3 and αvβ5 integrins are expressed on endothelial cells and can contribute to fibrosis by promoting angiogenesis and influencing HSC activation. Additionally, the activation of latent TGF-β through αv-containing integrins represents a central mechanism linking inflammation to fibrogenesis. Multiple cell types other than HSCs express αv integrins, including αvβ6 on injured cholangiocytes (Figure 6) and αvβ8 on dendritic cells [48]. These integrins can mechanically activate cytokines through force-dependent conformational changes by binding the RGD motif in the LAP element of latent TGF-β, as described above. Notably, in human cirrhosis and experimental models of biliary fibrosis, αvβ6 is overexpressed in cholangiocytes [133,134]. This overexpression is correlated with the severity of fibrosis. Pharmacological inhibition of αvβ6 has been shown to suppress cholangiocyte proliferation and mitigate liver fibrosis progression in preclinical studies [134]. Furthermore, the deletion of αvβ6 in a BDL mouse model resulted in reduced periodontal fibrosis [134,135]. These findings underscore the importance of αvβ6 in the fibrogenic response [35]. Its presence on activated biliary epithelial cells further suggests that αvβ6 could be a potential therapeutic target for managing biliary diseases [134,136,137].

## 5. Integration of Mechanosensitive Signaling Pathways in Liver Fibrosis

Recent advances in understanding liver fibrosis have revealed extensive crosstalk between major mechanosensitive signaling pathways, such as the Hippo/YAP, Rho/ROCK/MRTF, and TGF-β pathways. This integration creates a complex signaling network that coordinates fibrogenic responses and presents both challenges and opportunities for therapeutic intervention.

Mechanical forces serve as central regulators of all three pathways. Mechanical stress can simultaneously activate these signaling cascades through integrin-mediated mechanotransduction, cytoskeletal rearrangements, and ECM-cell feedback loops. RhoA/ROCK signaling plays a particularly important role by influencing YAP nuclear translocation through F-actin organization and by simultaneously activating MRTF via changes in the G-actin/F-actin ratio. TGF-β signaling integrates with these pathways through its ability to activate RhoA. Research suggests that sustained cytoskeletal changes may lead to long-term consequences of acute mechanical activation, highlighting the concept of mechanical memory.

At the transcriptional level, recent studies have revealed extensive cooperation between these pathways in cancer, with some evidence that this also applies to fibrosis [49,138,139,140,141,142]. YAP/TAZ and MRTF share common regulatory elements and cooperatively regulate fibrogenic genes through mechanosensitive enhancers [143]. TGF-β signaling mediates crosstalk with these mechanosensitive transcription factors by forming SMAD-containing complexes and regulating shared profibrogenic target genes (for example, *ACTA2*, *COL1A1,* and *CTGF*) [50,78,79,144]. These transcription factor complexes may assemble in a context-dependent manner, allowing flexible responses to different fibrogenic stimuli.

The integration of these pathways creates multiple feedback mechanisms that can amplify fibrogenic responses. Matrix-mediated feedback loops are particularly important, as pathway activation promotes ECM production, leading to increased tissue stiffness, increased integrin signaling, and increased mechanotransduction. At the molecular level, TGF-β increases the expression of integrin subunits, Rho guanine nucleotide exchange factors (GEFs), and YAP/TAZ target genes [144]. Conversely, YAP/TAZ activation enhances the expression of TGF-β signaling components, ROCK, and ECM protein and integrin production. In addition, MRTF upregulates the expression of TGF-β, thereby amplifying its profibrotic signaling [88].

Disease progression shows stage-specific patterns of pathway activation. Early fibrosis is characterized predominantly by YAP/TAZ activation, whereas progressive disease involves increasingly coordinated pathway activation. The advanced stages show sustained activation of all pathways. Spatial transcriptomics has further revealed zone-specific pathway activation patterns, the formation of activation hotspots, and gradient responses to mechanical stress [145,146,147].

These novel insights into pathway integration have important therapeutic implications. The development of combination therapy approaches may show promise due to synergistic effects. On the basis of the temporal and spatial patterns of pathway activation, stage-specific and fibrotic niche-specific intervention strategies should be considered to increase therapeutic effects while minimizing side effects. Furthermore, pathway integration signatures are emerging as valuable prognostic indicators, potentially enabling better monitoring of pathway activation and improved patient stratification strategies.

## 6. Therapeutic Approaches Targeting Mechanosensitive Signaling in Liver Fibrosis

Significant advances in therapeutic strategies targeting mechanosensitive pathways in liver fibrosis have been made in recent years. These strategies range from single-pathway inhibition to multi-pathway approaches. There is an increasing focus on more selective inhibition strategies that improve target specificity while reducing systemic effects. Here, we review the current status of select preclinical and clinical agents. Several reviews provide in-depth coverage of recent developments in targeting integrins in general and in fibrosis [148,149,150].

### 6.1. Pan-Integrin Inhibitors

Early therapeutic approaches focused on broad inhibition of β1 and αv integrins. These integrins are particularly attractive targets, as they are widely involved in fibrosis, and their disruption via antibody or chemical therapy prevents profibrotic molecular and cellular events in preclinical models. Because the ubiquitous expression of β1 integrins has raised concerns about potential off-target effects, early development focused on panαv therapeutic strategies. A variety of anti-αv antibody therapeutics and several peptide approaches that mimic RGD ligand binding have been developed and tested.

CWHM-12, a small-molecule RGD peptidomimetic compound that blocks αv-containing integrins involved in activating latent TGF-β, has significant antifibrotic effects on multiple organs, including the liver. Preclinical studies in mouse models of fibrosis (e.g., liver or lung) have shown reduced tissue scarring when CWHM-12 is administered. However, while this compound provides crucial proof-of-concept for targeting αv integrins in fibrosis, it has not advanced to clinical trials.

Cilengitide, a cyclic RGD pentapeptide that targets αv integrins (particularly αvβ3 and αvβ5), was one of the first integrin inhibitors to enter clinical development [151]. While primarily developed for cancer, preclinical studies in liver fibrosis have shown promise through the reduction of hepatic stellate cell activation. However, its short half-life and limited efficacy in clinical trials for other indications have limited its further development.

VPI-2690B is a first-in-class human monoclonal antibody developed by Vascular Pharmaceuticals for the treatment of diabetic nephropathy. VPI-2690B specifically targets αvβ3 and has shown promise in preclinical studies of diabetic nephropathy in phase I trials. However, challenges in further clinical development may have been encountered, as the latest update indicates a phase 2a trial (NCT02251067) to be completed in 2017. However, no results have been reported.

Thus, while targeting αv and β1 integrins has proven highly effective in reducing profibrotic signaling and fibrogenesis in preclinical studies, substantial concerns remain regarding the safety and toxicity of drugs targeting these biomolecules or their efficacy. Therefore, such strategies are unlikely to be translated to the clinic because of the widespread expression of these integrins and the potential for off-target effects.

### 6.2. Selective Single and Dual Integrin Inhibitors

More recent approaches have focused on selectively targeting specific integrin heterodimers, including αvβ6 and αvβ1, and the combined inhibition of αvβ6 and αvβ1 integrins due to their roles in activating the latent form of TGF-β.

A promising antifibrotic agent is the anti-αvβ6 humanized monoclonal antibody BG00011 (formerly STX-100), which is a first-in-class humanized anti-αvβ6 IgG1 monoclonal antibody developed by Biogen Idec (Cambridge, MA, USA). This antibody inhibits the binding of αvβ6 to the latent form of TGF-β, thereby preventing the activation of latent TGF-β1 [152,153]. BG00011 has undergone phase 2 clinical trials as a therapy for idiopathic pulmonary fibrosis (IPF) [154]. However, its development was halted in 2019 because of safety concerns (scarring of the lungs).

GSK3008348, an inhaled small molecule αvβ6 integrin inhibitor, was developed by GlaxoSmithKline (GSK) for IPF treatment. While a phase 1 trial (NCT02612051) initially showed good safety, tolerability, and pharmacokinetic profiles in healthy volunteers [155,156], GSK abandoned its further development after phase 2a trials. GSK3335103, another orally active, nonpeptide αvβ6 integrin inhibitor, was also developed by GSK, and several optimized derivatives of GSK333510 and GSK3008348 are currently in preclinical development [157].

PLN-1474 is a selective inhibitor of αvβ1 integrin that targets the activation of TGF-β1 (Figure 6, Table 1). Developed by Pliant Therapeutics in collaboration with Novartis for the treatment of NASH, this inhaled inhibitor is presently considered phase 2-ready. It demonstrated excellent safety and pharmacokinetic profiles in phase 1 trials. However, owing to a change in company strategy, Novartis terminated its partnership with Pliant Therapeutics in 2024.

PLN-74809 (Bexotegrast) is a small-molecule, orally administered, dual-selective inhibitor of αvβ6 and αvβ1 integrins that was also developed by Pliant Therapeutics (Figure 6, Table 1). These integrins play a role in activating latent TGF-β. Bexotegrast is currently undergoing phase 2 clinical trials for the treatment of IPF and primary sclerosing cholangitis (PSC). To date, a favorable safety profile and efficacy have been demonstrated in these trials [158,159,160,161,162]. The phase 2a (INTEGRIS-IPF) and 2b (BEACON-IPF) trials for IPF showed dose-dependent effects on forced vital capacity (FVC) and quantitative lung fibrosis imaging, as well as a reduction in biomarkers of fibrosis in lung fluid cells after 7 days of treatment. A phase 2a study (INTEGRIS-PSC) in PSC patients confirmed the potential of this drug to reduce fibrosis and inflammation in patients with liver diseases, including PSC. Reported outcomes included decreased scarring and tissue stiffness in the liver, improved liver blood tests, enhanced liver scans, and a reduction in cholangitis episodes. Bexotegrast has recently been granted fast-track designation and orphan drug designation by the FDA for IPF. Additionally, it received an Orphan Drug Designation from both the FDA and EMA for PSC. Ongoing clinical trials focus on further efficacy and safety testing in patients with IPF and PSC. Overall, the dual inhibition strategy may offer advantages over single integrin targeting, particularly in complex fibrotic diseases such as NASH.

### 6.3. TGF-β Pathway Inhibitors

TGF-β is an important therapeutic target in a variety of human diseases. Therefore, many attempts have been made to inhibit TGF-β activation via various approaches, such as inactivating anti-TGF-β antibodies, molecules that interfere with the interaction of latent TGF-β with integrins for its activation, or chemical inhibition of downstream effectors of the TGF-β signaling cascade. However, many approaches that target global TGF-β signaling have not been applied to human patients because of potential serious side effects such as cancer or immune dysregulation.

Pirfenidone was approved by the FDA for the treatment of IPF. The effects of pirfenidone are mediated, in part, through the inhibition of TGFβ synthesis and downstream TGF-β1–associated signaling (Figure 6, Table 1) [163]. Pirfenidone also reduces inflammatory responses by lowering the levels of TNFα, IL-1, IL-6, and IFNγ and preventing NFκB activation [163]. Post-marketing real-world analyses revealed persistent long-term effects of pirfenidone: pirfenidone exhibited significant antifibrotic effects in early-stage fibrosis and was associated with a reduced rate of FVC decline and increased patient survival [163]. However, the drug was less effective in advanced fibrosis and was not protective against disease progression. Additionally, pirfenidone is associated with a range of systemic side effects [163].

An aerosolized form of pirfenidone delivered via nebulization, prolonged-release pirfenidone (PR-PFD), is currently used for the treatment of IPF (approved by the FDA) and cirrhosis (approved in Mexico by COFEPRIS) [164,165] and is in early-phase trials for other applications (Figure 6, Table 1).

With a similar goal of attenuating systemic side effects while maintaining therapeutic effects, LYT-100, a deuterated form of pirfenidone, is currently being evaluated in clinical trials (Table 1). Recent results from a phase 2b trial (ELEVATE IPF), a randomized, double-blind, active and placebo-controlled, dose-ranging trial, revealed that LYT-100 effectively slowed lung function decline in patients with IPF, as measured by FVC, and achieved primary and key secondary endpoints. Notably, LYT-100 was evaluated alongside pirfenidone and not only outperformed pirfenidone but also demonstrated a more favorable tolerability profile compared with a similar dose of pirfenidone.

### 6.4. Targeting the Rho/ROCK/MRTF Pathway

#### 6.4.1. ROCK Inhibitors

The clinical development of ROCK inhibitors for liver fibrosis is still in relatively early stages. Most compounds are in preclinical or early clinical phases for the treatment of fibrotic diseases. Fasudil, a pan-ROCK inhibitor, was the first ROCK inhibitor to be clinically approved and is used for the treatment of cerebral vasospasm in Japan and China [166]. As a pan-ROCK inhibitor, fasudil lacks selectivity between ROCK1 and ROCK2. In addition, fasudil inhibits other kinases, such as PKA, PKG, PKC, and MLCK [167]. While fasudil pan-ROCK inhibition has shown therapeutic potential in various other diseases [167,168], the trend is moving toward selective ROCK2 inhibitors owing to their potentially improved safety profile. Selective ROCK2 inhibition may prevent the hypotensive side effects typically associated with systemic pan-ROCK inhibitors [166].

Zelasudil (RXC007), developed by Redx Pharma, is a potent and highly selective ROCK2 inhibitor in clinical development for IPF (Table 1). While its primary focus is on idiopathic pulmonary fibrosis (IPF), preclinical studies have shown promising results for treating other fibrotic indications, including liver fibrosis. A phase 2a study in IPF was recently completed as of Q4 2024.

Several other ROCK inhibitors have been evaluated, including KD025 (belumosudil) and GV101. Belumosudil, a specific inhibitor of ROCK2 [169], was recently approved by the FDA for the treatment of graft-versus-host-disease (GVHD), particularly its fibrotic component, after the failure of prior therapies [170,171]. Preclinical studies suggest that belumosudil has promise for treating fibrotic symptoms, including liver fibrosis [169,172], indicating its potential broader application beyond GVHD [173]. GV101 is another highly selective ROCK2 inhibitor with over 1000-fold selectivity for ROCK2 over ROCK1 [169]. It has shown significant promise in treating liver fibrosis in preclinical studies. GV101 treatment attenuated established liver fibrosis induced by thioacetamide in combination with a high-fat diet in mice, reduced collagen levels in the liver, improved liver function markers, and demonstrated anti-inflammatory and antimetabolic effects on human monocytes and Kupffer cells [169]. As research progresses, these ROCK inhibitors could provide new therapeutic options for patients with liver fibrosis.

#### 6.4.2. MRTF Inhibitors

Multiple MRTF inhibitors are currently undergoing preclinical development for potential therapeutic use. These inhibitors prevent MRTF nuclear localization and its interaction with SRF, thus inhibiting MRTF/SRF-mediated transcription linked to fibrosis and cancer progression [174]. CCG-1423 is a first-generation Rho/MRTF/SRF pathway inhibitor developed by researchers at the University of Michigan. It has shown efficacy in various antifibrotic activities across multiple tissues, including dermal, cardiac, lung, liver, and intestinal fibrosis [56,175,176,177,178,179]. CCG-1423 decreased matrix stiffness and TGF-β-induced fibrogenesis in vitro and in vivo, although it exhibited unacceptable cytotoxicity.

Second-generation MRTF/SRF inhibitors with improved safety and efficacy profiles have been developed. Notable examples include CCG-203971 and CCG-222740, which are advancing through preclinical development [179,180,181,182,183,184]. For example, CCG-222740 is reported to be five times more potent than CCG-203971 in inhibiting MRTF/SRF target genes and effectively reduces scar tissue formation in fibrosis models while exhibiting lower cytotoxicity than earlier inhibitors do [185]. Recently discovered by Cincera Therapeutics and Monash University, CIN-244 is another MRTF/SRF pathway inhibitor, primarily designed to treat fibrotic diseases, which is currently still in preclinical evaluation.

### 6.5. Targeting YAP/TAZ

The YAP pathway is a strategic target not only in fibrosis but also in cancer. The first identified YAP inhibitor, verteporfin, has shown promising preclinical results but has stalled in early clinical development owing to potential off-target effects and unfavorable pharmacokinetics.

Recent advances in YAP/TAZ pathway inhibition have produced several candidates in both preclinical and clinical stages. The focus is currently on understanding their efficacy across various cancer types, particularly those characterized by Hippo pathway dysregulation. Notably, direct inhibitors now target the YAP–TEAD interaction with high selectivity and potency. One example is IAG933, which directly disrupts the YAP–TEAD interaction, inhibiting YAP–TEAD-dependent transcription and leading to significant tumor regression [186]. This compound has shown promising preclinical results, including significant tumor regression in models of mesothelioma and other cancers, and is currently in phase I clinical trials. This agent is currently also undergoing clinical evaluation, and combinations of IAG933 with KRAS inhibitors have demonstrated enhanced antitumor effects in pancreatic and colorectal models, potentially overcoming treatment resistance [186].

Allosteric inhibitors present another approach by binding to specific sites on TEAD proteins. For example, GNE-7883 is a potent small-molecule inhibitor that blocks interactions between YAP/TAZ and all human TEAD paralogs. This compound has demonstrated effectiveness in disrupting the oncogenic transcriptional program linked to YAP/TAZ [187]. Other allosteric inhibitors, such as VT104, K-975, and SWTX-143, target the lipid pocket of TEAD and exhibit varying potency across TEAD paralogs [188,189,190,191]. Thus, while YAP inhibitors are being developed primarily for cancer therapy, particularly for mesothelioma, they are likely promising for treating fibrosis given the critical role of the Hippo/YAP pathway in this disease.

In summary, the development of integrin-targeted therapies for liver fibrosis continues to evolve, with several promising approaches in various stages of development. The development of downstream components of pathways activated by integrins and these inhibitors reflects ongoing efforts to develop new targets for therapeutic benefits in fibrosis. The challenges faced by some candidates, particularly regarding safety and efficacy, highlight the importance of careful target validation and patient stratification in future clinical trials. As observed with Bexotegrast, combination approaches that target multiple components may offer more effective therapeutic strategies.

## 7. Future Perspectives

The role of integrins in liver fibrosis has gained significant attention in recent years, driven by the unmet need for effective antifibrotic therapies for chronic liver diseases. At the heart of this interest is the dual capacity of integrins to both sense and shape the extracellular environment, functions that are central to hepatic fibrogenesis. While the past decade has yielded key insights into how integrins such as αvβ1, αvβ6, αvβ8, and other integrins regulate HSC activation, immune cell recruitment, and TGF-β signaling, several critical questions remain. Addressing these gaps will likely pave the way for the development of more efficient therapeutic interventions with limited side effects.

One of the most pressing challenges in understanding liver fibrosis is delineating the context-specific functions of integrins across different disease stages and etiologies. For example, chronic hepatitis B, autoimmune hepatitis, and NASH each involve unique molecular triggers and patterns of immune infiltration, which may distinctly modulate integrin expression and activation. Elucidating the roles of specific integrins and their downstream effectors in profibrotic signaling across these varied contexts is essential for designing targeted therapies that minimize off-target effects while enhancing efficacy. For example, blocking the action of certain integrins on inflammatory cells may be more effective in conditions driven by immune-mediated injury, whereas targeting integrin-dependent mechanotransduction may be more beneficial in conditions where ECM remodeling and increased tissue stiffness are prominent features, as is often the case in NAFLD/NASH.

Furthermore, targeting multiple integrin subtypes or downstream pathways might be necessary for effective antifibrotic therapy. Towards this aim, the crosstalk between integrins, MRTF, YAP/TAZ, and TGF-β activation in HSCs and other liver-resident cells, as well as their individual contribution to fibrogenesis, warrants further investigation. In particular, a deeper understanding of the spatiotemporal regulation of signaling processes and the extent of redundancy during progressive fibrogenesis is needed. Another important area for future research is the interplay between integrins and the immune microenvironment in the fibrotic liver. Immune cells, including macrophages, lymphocytes, and other populations, rely on integrins, such as those in the β1 and β2 families, for tissue infiltration and retention. The extent to which HSCs or other resident cells, like sinusoidal endothelial cells, modulate integrin ligation to influence immune cell function (and vice versa) remains to be fully explored. Addressing this gap could illuminate key mechanisms underlying both the initiation and resolution phases of fibrogenesis, particularly given emerging evidence that distinct myeloid and lymphocyte subsets can either exacerbate or mitigate fibrotic progression depending on local integrin signaling cues. Recent advances in single-cell and proteomic approaches are beginning to offer detailed insights into the spatiotemporal dynamics of the initial injury response, fibrosis development, and regression across different liver disease etiologies [145,146,147,192,193,194,195,196]. These technologies are also enhancing our understanding of myofibroblast heterogeneity and the complex crosstalk between myofibroblasts and immune cells.

Additionally, integrins serve not only as signaling receptors but also as critical mediators of cellular responses to stiffness in the fibrotic liver. In progressive fibrosis, liver tissue undergoes marked increases in matrix stiffness that, in turn, modulate integrin activation in the fibrotic niche. Employing technologies such as atomic force microscopy and 3D hydrogel-based culture systems that mimic physiological stiffness gradients will likely enhance our understanding of these mechanosensitive pathways and their roles in fibrosis progression. A better understanding of the mechanobiology of liver fibrosis may also lead to the development of new strategies to achieve improved tissue specificity of integrin inhibition, potentially through advanced drug delivery systems, such as mechanically responsive nanoparticles, hydrogel-based carriers, or integrin-directed ligand–drug conjugates.

From a translational perspective, integrin-targeting strategies have already shown promise in clinical studies, yet many fundamental questions remain regarding their optimization. For instance, systemic inhibition of specific integrins like αvβ6 might lead to unexpected adverse effects, given the broad physiological roles of these molecules beyond the liver. The inherent complexity and potential redundancy of integrin-mediated processes present both challenges and opportunities. It is conceivable that effective antifibrotic therapy will require targeting multiple integrin subtypes (as shown for Bexotegrast) and/or downstream pathways. Future studies should, therefore, prioritize strategies to enhance localized targeting effects while reducing off-target toxicity. At the same time, the disease-specific involvement of integrins suggests that tailored therapeutic approaches could be developed for different etiologies of liver fibrosis.

Finally, a critical frontier for future research lies in establishing the long-term clinical benefits and safety profiles of integrin-based therapies in liver disease patients. Pilot studies should investigate whether combinatorial regimens, such as pairing integrin inhibitors with anti-inflammatory or metabolic treatments, can provide synergistic antifibrotic benefits. Rigorous clinical trial designs, along with robust biomarker discovery, will be essential for monitoring patient responses, optimizing dosing, and minimizing on-target/off-target complications, especially given the fundamental roles of integrins, TGF-β, and mechanosensitive downstream signaling pathways in maintaining organ and tissue homeostasis beyond the liver.

## 8. Conclusions

It has become increasingly evident that integrins and ECM stiffness profoundly influence fibrosis across multiple organs. Within the liver, integrins and mechanosensitive signaling pathways are central to tissue fibrogenesis, as they contribute to the activation of HSCs, the primary drivers of fibrosis, and provide crosstalk with immune cells. Moving forward, therapeutic strategies aimed at targeting integrins or their downstream signaling components, whether through antibody blockade or small-molecule inhibition, hold great promise for the development of effective antifibrotic therapies. By expanding our knowledge of integrin specificity, signal integration with TGF-β and other pathways, mechanobiological feedback loops, and crosstalk with the microenvironment, we will move closer to translating these new insights into innovative treatments. Continued efforts in fundamental research and carefully designed clinical trials will be indispensable for achieving tangible breakthroughs for patients with chronic liver disease.

## Figures and Tables

**Table 1:** Select inhibitors of integrins and downstream signaling components relevant to fibrosis.

	Mechanism of Action	Stage of Evaluation	ClinicalTrials.gov Identifier
PLN-74809 (Bexotegrast)	PLN-74809 is an oral small-molecule dual inhibitor of αvβ1 and αvβ6 integrins. Developed by Pliant Therapeutics. By inhibiting these integrins, it aims to prevent the activation of latent TGF-β1 and TGF-β3, reducing fibrosis.	PLN-74809 is in two phase II clinical trials targeting IPF and primary sclerosing cholangitis (PSC). Clinical data suggest it is well-tolerated and reduces biomarkers of fibrosis. Bexotegrast has been granted fast track designation and orphan drug designation by the FDA for IPF. Additionally, it received Orphan Drug Designation from both the FDA and EMA for PSC.	04480840 04072315 05621252 04396796
PLN-1474	PLN-1474 is a selective inhibitor of αvβ1 integrin, targeting the activation of TGF-β1 in the liver to reduce fibrosis. Developed by Pliant Therapeutics, in collaboration with Novartis	PLN-1474 is has completed phase I clinical trials for liver fibrosis associated with NASH.	Not reported
Pirfenidone (PFD)	Inhibition of TGFβ synthesis and activation of downstream signaling. Anti-inflammatory effects through downregulation of TNFα, IL-1 and IL-6, IFNγ along with NFκB inactivation among other effects.	Approved by FDA for IPF.	
PR-PFD	Prolonged-release pirfenidone, administered via nebulizer	Approved by FDA for IPF and in Mexico approved by COFEPRIS for advanced liver fibrosis.	05542615
LYT-100	Deuterated form of pirfenidone (PureTech Health plc)	Successful Phase 2b Trial for IPF concluded.	05321420
AVID200	AVID200 is a TGF-β ligand trap engineered to selectively inhibit TGF-β1 and TGF-β3 isoforms while sparing TGF-β2, aiming to reduce fibrosis with fewer side effects.	In early clinical phase I trials for fibrotic diseases, including systemic sclerosis (SSC) and myelofibrosis (MF). Studies have shown promising results warranting potential applications in other fibrotic conditions.	03831438, 03895112
Zelasudil (RXC007)	Zelasudil, developed by Redx Pharma, is a potent and highly selective ROCK2 inhibitor in clinical development for IPF.	Preclinical studies have shown promising results for treating other fibrotic indications, including liver fibrosis. Completed a Phase 2a study in IPF as of Q4 2024.	Not reported

Note: This table primarily includes compounds that have reached clinical development specifically for fibrotic conditions.

## Data Availability

Not applicable. No new data were generated.
